# Blastic plasmacytoid dendritic cell neoplasm and cerebral toxoplasmosis: a case report

**DOI:** 10.1186/s12883-022-02748-5

**Published:** 2022-06-24

**Authors:** Anna Maria Florescu, Anne Louise Tølbøll Sørensen, Henrik Vedel Nielsen, Daniel Tolnai, Lene Dissing Sjö, Katja Lohmann Larsen, Mohammad Al-Mahdi Al-Karagholi

**Affiliations:** 1 Department of Neurology, Rigshospitalet Glostrup, Faculty of Health and Medical Sciences, University of Copenhagen, Glostrup, Denmark; 2 Department of Hematology, Rigshospitalet, Faculty of Health and Medical Sciences, University of Copenhagen, Copenhagen, Denmark; 3 Laboratory of Parasitology, Department of Bacteria, Parasites & Fungi, Infectious Disease Preparedness, Statens Serum Institut, Copenhagen, Denmark; 4 Department of Diagnostic Radiology, Rigshospitalet Glostrup, Faculty of Health and Medical Sciences, University of Copenhagen, Glostrup, Denmark; 5 Department of Pathology, Rigshospitalet, Faculty of Health and Medical Sciences, University of Copenhagen, Copenhagen, Denmark

**Keywords:** Headache, Confusion, Hematologic malignancy, Acute Leukemia, Cerebral toxoplasmosis

## Abstract

**Background:**

The present case contributes to the limited literature on central nervous system involvement of blastic plasmacytoid dendritic cell neoplasm (BPDCN).

**Case presentation:**

A 63-year-old male presented to the department of neurology with a three-day history of rapidly progressing headache, fatigue, and confusion. Physical examination revealed multiple bruise-like skin lesions. Initial laboratory workup raised suspicion of acute leukemia, and a brain computer tomography identified several hyperdense processes. A bone marrow biopsy gave the diagnosis BPDCN, a rare and aggressive hematologic malignancy derived from plasmacytoid dendritic cells with a poor prognosis. Lumbar puncture showed not only signs of BPDCN, but also cerebral toxoplasmosis, thus providing a differential diagnosis. Despite intensive systemic and intrathecal chemotherapy, the patient died 25 days later due to multi-organ failure.

**Discussion:**

The exact incidence of BPDCN is unknown and perhaps underestimated but may account for 0.5 – 1% of all hematological malignancies. The median age at onset is 60 to 70 years, and most patients are men. Cutaneous lesions are the most frequent clinical manifestation at diagnosis. Other symptoms present at time of diagnosis or during disease progression include lymphadenopathy, splenomegaly and cytopenia caused by bone marrow involvement. Although the majority of BPDCN patients have no symptoms or signs of central nervous system involvement, plasmacytoid dendritic cells have been detected in the cerebrospinal fluid in more than 50%.

**Conclusions:**

This case highlights the importance of considering hematological malignancies as a differential diagnosis in patients developing acute neurological symptoms and raises suspicion of a possible association between toxoplasmosis and hematological malignancies.

## Background

Blastic plasmacytoid dendritic cell neoplasm (BPDCN) is a rare hematologic malignancy with a poor prognosis and approximately 1000 cases in Europe per year [1]. About 75% cases of BPDCN occur in men and median age at onset is 61 to 67 years [2]. Clinically, BPDCN is an aggressive tumor derived from the precursors of plasmacytoid dendritic cells and often presents with cutaneous manifestations and involvement of bone marrow, lymph nodes and peripheral blood [3]. Involvement of the central nervous system (CNS) has been reported with variable frequency [4–7], but not as the presenting symptom. Here, we report an atypical presentation of BPDCN with rapidly developing neurological symptoms as the main reason for hospitalization and a concomitant diagnosis of cerebral toxoplasmosis.

## Case presentation

**Fig. 1:**
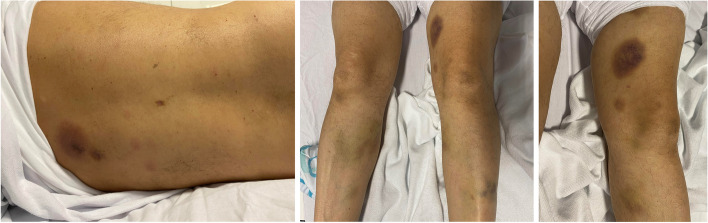
Cutaneous lesions

**Fig. 2:**
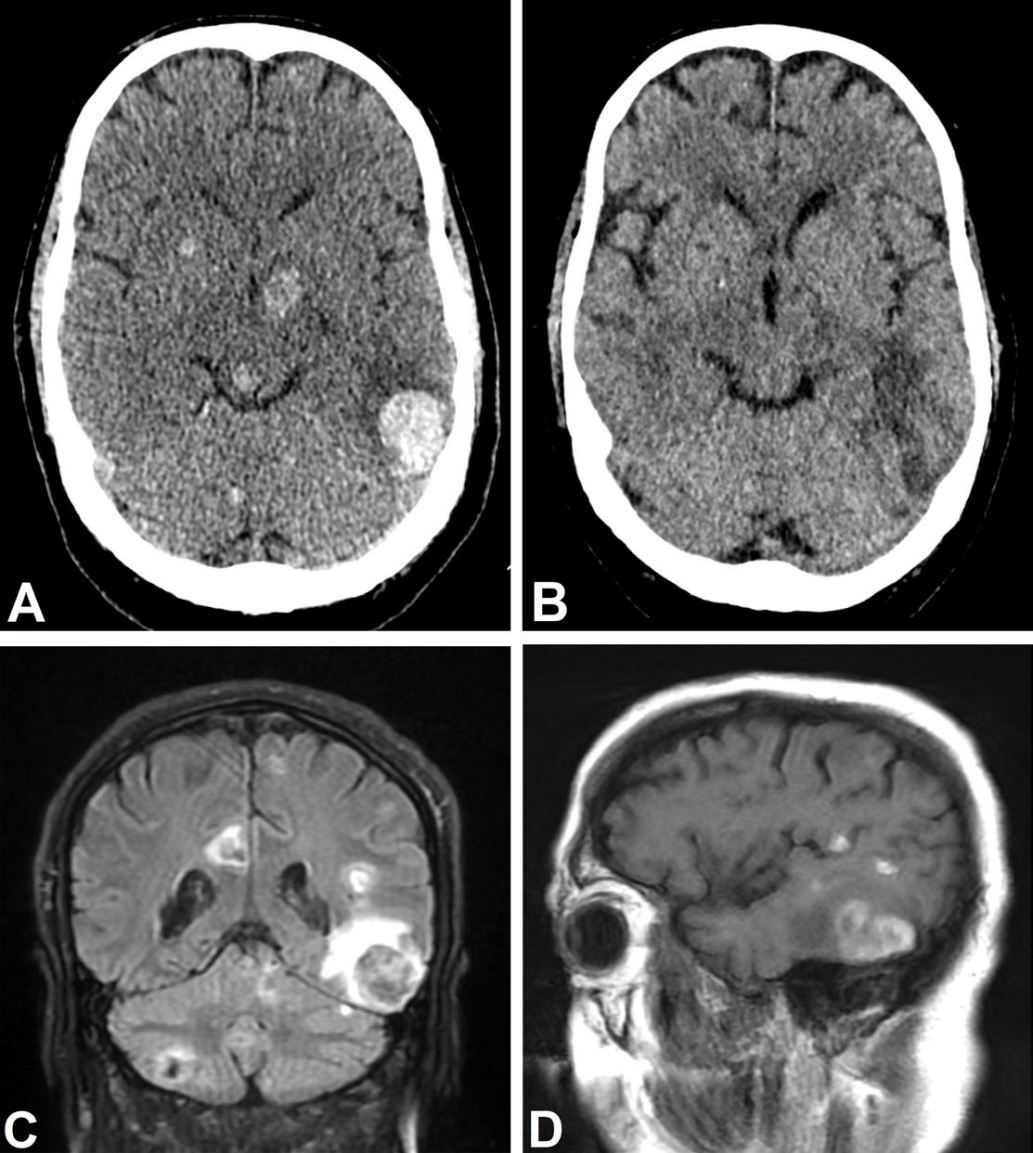
Brain imaging. **A**. A 0.62 mm slice of the initial non-contrast CT-head. The first scan is performed on the day of admission showing multiple rounded hyperdensities in supra- and infratentorial regions. The largest lesion is in the in the left temporal lobe and measures 34 × 25 mm in the axial plane. Slight edema surrounds the lesions but has restricted local mass effect. The hyperdensities indicate hemorrhages, tentative diagnoses are metastases or cavernous malformations due to the scattered pattern. **B**. A 0.62 mm slice from the last non-contrast CT-head performed 30 days after initial scan, reconstructed approximately at the same plane and incline angle. It shows a significant decrease of the hyperdensities as well as a small lesion with loss of substance in the right putamen, both indicating partial resorption of hemorrhages. **C**. Coronal T2 weighted FLAIR image from the single MRI-head performed one day after admission. It shows the same scattered lesions both supra- and infratentorially in both hemispheres. The largest lesion is also seen in the left temporal lobe with centrally heterogeneous signal values suggestive of hematoma. **D**. Sagittal T1 weighted FLAIR image showing high signal in the border zone of the lesions further strengthening the suspicion of bleeding/hematomas. This and the subsequent MRI sequences were marred by movement artefacts and besides DWI no further sequences of diagnostic value could be made

A 63-year-old man with a previous spinal disc herniation and no current medication presented to the department of neurology with headache, nausea, fatigue, and concentration difficulties through three days. The headache was intermittent, diffuse, and pressing, 4–5 on the numerical rating scale (NRS 0–10) with a single episode of emesis and no specific triggers. The patient appeared lethargic and was not able to speak in coherent sentences, but no aphasia was observed. Further neurological examination was normal, and the patient showed no signs of respiratory or cardiovascular failure with normal vital signs except for a blood pressure of 195/103 mm Hg. Skin inspection revealed localized purplish macules measuring up to 5 cm in diameter on arms, legs, and torso, persisting for two weeks with no prior history of trauma (Fig. 1). Laboratory workup showed significant leukocytosis, moderate thrombocytopenia, normal hemoglobin, and elevated lactate dehydrogenase with no signs of disseminated intravascular coagulation (DIC) or tumor lysis (Table 1). A computer tomography (CT) of the brain without intravenous contrast showed multiple hyperdense processes throughout the supra- and infratentorial regions with the largest in the left temporal lobe. The processes were initially interpreted as hemorrhages, tentative diagnoses being metastases or cavernous malformations (Fig. 2A).

**Table 1:** Blood tests

	**November 12th**	**November 23rd**	**Decmber 12th**
Hemoglobin	13.4 g/dL	-	8.5 g/L
Erythrocytes	5.1 E12/L	-	-
Leukocytes	97.2 E9/L	-	< 0,10 E9/L
Platelets	48 E9/L	-	4 E9/L
Blast	78.7 E9/L	0.16 E9/L	-
Lymphocytes	11 E9/L	0.6 E9/L	-
Lactate dehydrogenase	1620 U/L	-	243 U/L
C-Reactive Protein	15 mg/L	-	370 mg/L
D-dimer	0.43 FEU/liter	-	-
Fibrinogen	18.4 μmol/liter	-	-
Antithrombin	0.70 IU/liter	-	-

The patient was immediately referred to the department of hematology under suspicion of acute leukemia. A leukocyte differential count demonstrated the presence of 79 × 10E9/L blasts in peripheral blood. Examination of a peripheral blood smear confirmed pronounced leukocytosis dominated by blastic cells with large round to slightly irregular nuclei and a greyish-blue cytoplasm without granules or Auer rods. Acute cytoreductive chemotherapy with cytarabine and supportive treatment was initiated given a tentative diagnosis of non-promyelocytic acute leukemia with hyperleukocytosis and a suspicion of leukostasis as a contributing factor to the neurological affection.

**Fig. 3:**
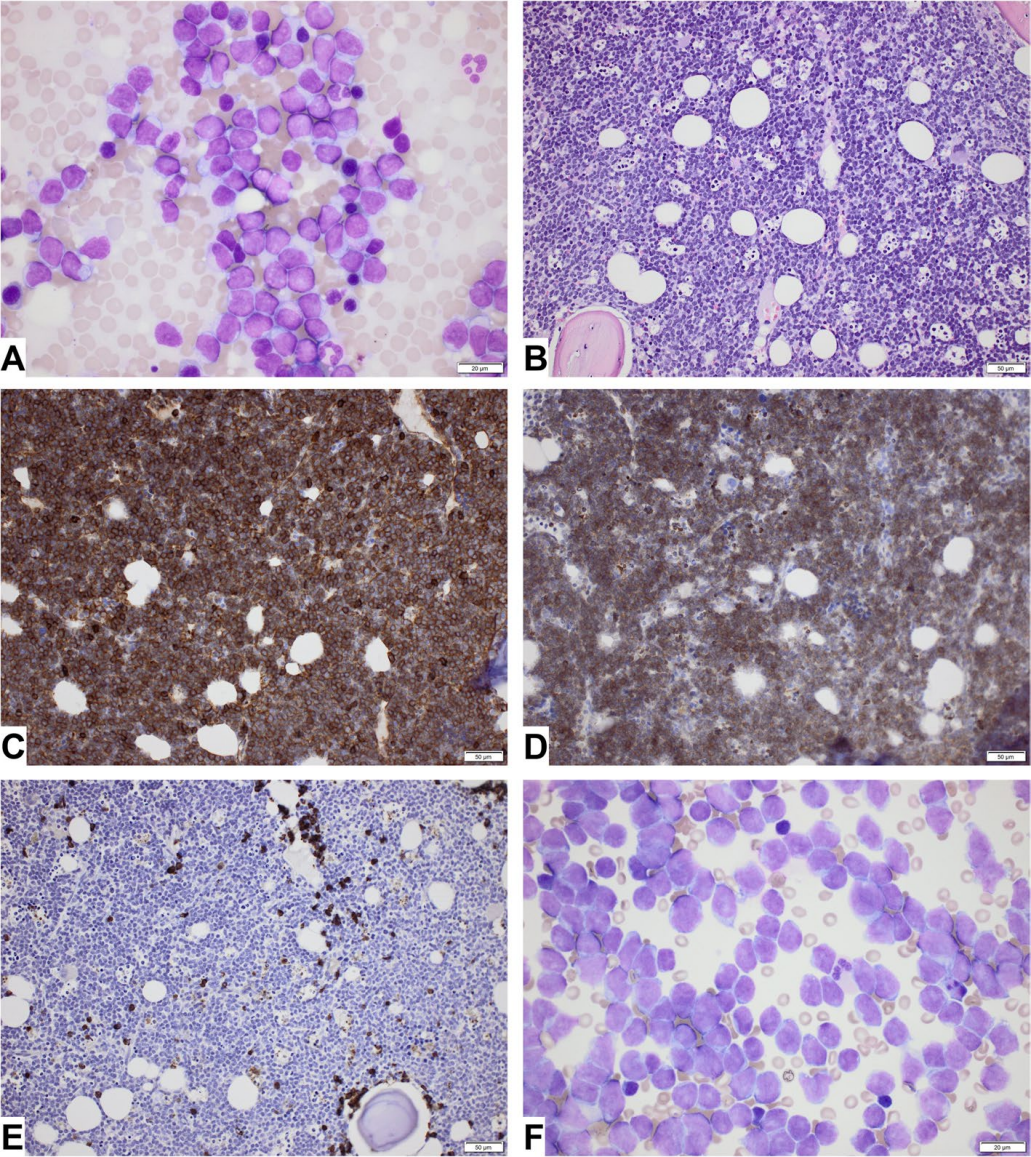
Histology. **A**. Imprint from bone marrow showing dominance of large blastic cells (× 60). **B**. Bone marrow trephine was hypercellular with dominance of blastic cells. The blastic cells were immunohistochemically positive in CD4 (**C**) and CD123 (**D**) (× 20). **E**. Lysozyme was positive in scattered myeloid cells, but negative in the blastic cells (× 20). **F**. CSF heavily infiltrated with blasts (× 60). Type of equipment for all microscopy images: microscope: Olympus BX53; objective: UPlanSApo; camera: Olympus UC30, U-TVO.5XC-3, SN5A00970; software: cellSens Entry

A magnetic resonance imaging (MRI) of the brain without intravenous contrast strengthened the suspicion of the processes being hematomas, but due to movement artefacts the images were suboptimal, and an affirmative underlying reason could not be determined (Fig. 2C and D). Intensive standard chemotherapy induction regimen with daunorubicin (60 mg/m2) and cytarabine (100 mg/m2) (DA 3 + 10) was initiated based on flow cytometry analysis (EuroFlow AML/MDS antibody panel tube 1–4) demonstrating 85% CD34 negative, CD117 negative, HLA-DR positive, MPO negative, CD13 negative, CD33 positive, CD56 variable, CD45 dim myeloid blasts [8]. Lumbar puncture demonstrated pronounced infiltration of blasts in the cerebrospinal fluid (CSF), and triple intrathecal therapy with hydrocortisone (16 mg), methotrexate (12 mg), and cytarabine (30 mg) was initiated. Pathological examination of the bone marrow biopsy using a more comprehensive immunohistochemical panel including CD4 and CD123 showed an extremely hypercellular marrow with severe dominance of blastic cells expressing CD4, CD123, CD33 and partly CD56 verified by immunohistochemistry. There was no expression of CD34, CD117, MPO, lymphoid- or monocytic markers (Fig. 3). Conclusively, the criteria for a diagnosis of blastic plasmacytoid dendritic cell neoplasm (BPDCN) were fulfilled and treatment with bortezomib (1 mg/kg) and intravenous dexamethasone (20 mg) was added.

Despite clearance of leukemic cells in the CSF and regression of the cerebral lesions on scans the patient’s cerebral status worsened with declining consciousness (Fig. 2B). Considering the immunosuppressive treatment alternative causes were examined. Blood serum was negative for herpes simplex virus and varicella zoster virus, cryptococcus antigens and echinococcus antibodies. CSF was also negative for herpes simplex virus, varicella zoster virus and cryptococcus antigens. In contrast, Toxoplasma gondii DNA was detected in the CSF by polymerase chain reaction (PCR) with a cycle threshold value of 30 (reference value 20 – 40) in a sample obtained 16 days after chemotherapy was initiated. In alignment with this, although not confirmatory for a reactivated infection, Toxoplasma gondii serology was negative for IgM and positive for IgG (58 international units), the latter with a high avidity (0.606), indicating primary infection took place several months or years earlier. No toxoplasma gondii DNA was detected in the bone marrow. Treatment with sulfadiazine, pyrimethamine and leucovorin was added. Twenty-five days after initiation of intensive systemic and intrathecal chemotherapy the patient developed multi-organ failure and was transferred to the intensive care unit but died the following day.

## Discussion and conclusions

The majority of patients with BPDCN present with skin manifestations seen as non-pruritic purplish nodular or bruise-like skin lesions [5]. Cytopenia due to bone marrow infiltration frequently occurs at diagnosis or disease progression. Other sites of involvement include the spleen, lymph nodes, and other extra-nodal tissues, of note often with spreading to CNS [4, 9, 2]. A study detected plasmacytoid dendritic cells in the cerebrospinal fluid in more than 50% of patients when examined by flow cytometry [9].

The diagnosis BPDCN relies on detection of the specific immunophenotype by immunohistochemistry or flow cytometry of peripheral blood, bone marrow, skin biopsy or other available material. Extended panels are required to detect the malignant cells that express CD4, CD56, and CD123. Additional markers restricted to plasmacytoid dendritic cells including CD303, TCF4, and TCL1A are recommended. BPCDN is typically negative for other lineage-specific markers, and negative for CD34 [10]. BPDCN has no definite consensus regarding treatment regimen. In younger patients (< 65 years or < 75 years without comorbidities) eligible for myeloablative allogeneic stem cell transplantation (allo-SCT) standard treatment is intensive induction chemotherapy, most often acute myeloid leukemia (AML)-like, acute lymphoid leukemia (ALL)-like, or lymphoma-like chemotherapy [11].

The present case is typical regarding sex and age, but illustrates an unusual clinical presentation with headache, fatigue, concentration difficulties and multiple cerebral lesions. Although prolonged unrecognized disease cannot be excluded, BPDCN can have a rapid course of CNS involvement regardless of clinical presentation and at any time of diagnosis [9]. While CNS involvement was proven by CSF flow cytometry analysis, it remains unclear whether the preliminary neurological condition and the findings on the CT- and MRI-scans can be attributed to cerebral dissemination of BPDCN, cerebral toxoplasmosis or both. Toxoplasmosis is a common parasite infection worldwide. A recent Danish study showed a seroprevalence of immunoglobulin G antibodies against Toxoplasma gondii in approximately 26% in a cohort with a mean age of 37,4 years [12]. Cerebral toxoplasmosis due to adequate immunosuppression is commonly the result of latent infection reactivation and presents with neurological symptoms such as headache, disorientation, hemiparesis, and seizures [13].

The brain lesions could represent cerebral toxoplasmosis due to their distribution and morphologic characteristics [14]. They were predominantly located at the basal ganglia, thalami and subcortically, which are common sites for toxoplasmosis (Fig. 2). Especially the largest lesion in the left temporal lobe had a heterogenous signal intensity on the T2 weighted FLAIR images with surrounding oedema, which is another typical finding. On the Diffusion Weighted Images (DWI) most lesions had a low signal in the center and high signal at the border zone indicating central necrosis. The largest lesion had some brighter spots centrally suggestive of more organized material as in an immature abscess. However, toxoplasmosis does not commonly cause hemorrhagic transformation, and unfortunately the signal on DWI is often confounded by bleeding in the lesions as in this case.

Due to the patients’ clinical condition, it was not possible to attain a contrast enhanced MR- or CT-head when the lesions were at their peak size. Sixteen days after initiation of intensive systemic and intrathecal chemotherapy, including high dose dexamethasone, CSF was positive for toxoplasma gondii; 23 and 24 days after treatment initiation a CT-head (with and without contrast, respectively) showed significant lesion regression and no enhancement was visible. Notably, the contrast enhanced CT was performed 2 days before toxoplasmosis treatment was initiated. In conclusion, a definitive underlying cause could not be determined with the scans. A biopsy of the cerebral lesions or a postmortem neuropathological examination was not conducted and hence no histopathological diagnosis obtained.

Interestingly, toxoplasma gondii infects and uses dendritic cells to spread the infection throughout the human body, and toxoplasmosis is furthermore considered a differential diagnosis to BPDCN [15, 16]. Whether this connection is of any significance to this case report remains unclear due to the limited literature within the field, but a possible association between toxoplasmosis and hematological malignancies has earlier been suspected [17].

It requires a multidisciplinary approach to diagnose and treat BPDCN [18]. Intensive chemotherapy results in a high response rate (40–80% in retrospective studies depending on the regimen used), but most patients relapse within a median time of 9 months, and overall survival is 12–18 months [11]. The most efficient chemotherapy regimens based on retrospective studies are AML-like and especially ALL-like regimens, however, consolidation with allo-SCT in first complete remission is required for long-term survival [19]. Pre-emptive intrathecal chemotherapy should be considered due to frequent CNS involvement and has been reported to prolong survival in BPDCN with CNS involvement at diagnosis [9]. Newer treatment modalities include combination with immunomodulatory drugs and targeted therapies, i.e. bortezomib, venetoclax and tagraxofusp (SL-401, a CD123-directed cytotoxin consisting of human interleukin-3 fused to truncated diphtheria toxin) [20]. Older patients ineligible for intensive chemotherapy can be treated with low intensity or palliative chemotherapy.

In conclusion this case contributes to the relatively limited literature on BPDCN with CNS involvement and a concomitant diagnosis of cerebral toxoplasmosis.

## Data Availability

Not applicable.

## Abbreviations

ALL: Acute lymphoid leukemia
allo-SCT: Allogeneic stem cell transplantation
AML: Acute myeloid leukemia
BPDCN: Blastic plasmacytoid dendritic cell neoplasm
CNS: Central nervous system
CSF: Cerebrospinal fluid
CT: Computer tomography
DIC: Disseminated intravascular coagulation
DWI: Diffusion Weighted Images
MRI: Magnetic resonance imaging
NRS: Numerical rating scale
PCR: Polymerase chain reaction
